# Assessing Brain Networks by Resting-State Dynamic Functional Connectivity: An fNIRS-EEG Study

**DOI:** 10.3389/fnins.2019.01430

**Published:** 2020-01-24

**Authors:** Yujin Zhang, Chaozhe Zhu

**Affiliations:** ^1^Brainnetome Center, Institute of Automation, Chinese Academy of Sciences, Beijing, China; ^2^National Laboratory of Pattern Recognition, Institute of Automation, Chinese Academy of Sciences, Beijing, China; ^3^State Key Laboratory of Cognitive Neuroscience and Learning, Beijing Normal University, Beijing, China

**Keywords:** functional connectivity, resting state, dynamic, functional near-infrared spectroscopy, electroencephalogram

## Abstract

The coordination of brain activity between disparate neural populations is highly dynamic. Investigations into intrinsic brain organization by evaluating dynamic resting-state functional connectivity (dRSFC) have attracted great attention in recent years. However, there are few dRSFC studies based on functional near-infrared spectroscopy (fNIRS) even though it has some advantages for studying the temporal evolution of brain function. In this research, we recruited 20 young adults and measured their resting-state brain fluctuations in several areas of the frontal, parietal, temporal, and occipital lobes using fNIRS-electroencephalography (EEG) simultaneous recording. Based on a sliding-window approach, we found that the variability of the dRSFC within any region of interest was significantly lower than the connections between region of interests but noticeably greater than the correlation between the channels with a short interoptode distance, which mainly consist of physiological fluctuations occurring in the superficial layers. Furthermore, based on a time-resolved *k*-means clustering analysis, the temporal evolution was extracted for three dominant functional networks. These networks were roughly consistent between different subject subgroups and in varying sliding time window lengths of 20, 30, and 60 s. Between these three functional networks, there were obvious time-varied and system-specific synchronous relationships. In addition, the oscillation of the frontal-parietal-temporal network showed significant correlation with the switching of one EEG microstate, a finding which is consistent with a previous functional MRI-EEG study. All this evidence implies the functional significance of fNIRS-dRSFC and demonstrates the feasibility of fNIRS for extracting the dominant functional networks based on RSFC dynamics.

## Introduction

The human brain is a highly complex network with dynamic and context-dependent coordination between disparate neural populations. Functional connectivity, which refers to the neural synchronization between brain areas, has been shown to reflect the information interactions between disparate neural populations. Based on the functional connectivity during the resting state (RSFC), researchers have produced a wealth of literature and revealed a great deal of information about the large-scale organization of the brain.

Although most RSFC studies are based on an implicit assumption that the brain functional connectivity is temporally stationary throughout the measurement period, rich evidence has amply confirmed that RSFC is highly non-stationary (Chang and Glover, 2010; Hutchison et al., 2013a; Mueller et al., 2013; Allen et al., 2014; Damaraju et al., 2014; Kucyi et al., 2017). In some cases, RSFC varies greatly between strongly positive and strongly negative correlations within time scales of seconds to minutes. Such temporal fluctuations of the dynamic RSFC (dRSFC) have been shown to be an essential property that can unveil flexibility in the dynamic functional coordination between different neural systems (Allen et al., 2014) and are not exclusive to humans (Majeed et al., 2011; Hutchison et al., 2013b; Keilholz et al., 2013). Furthermore, disruptions in RSFC dynamic characteristics are evidence of abnormal brain activity in mental disorders, such as schizophrenia (Damaraju et al., 2014; Zhang et al., 2016; Sanfratello et al., 2019), depression (Demirtas et al., 2016), attention deficit hyperactivity disorder (Zhang et al., 2016), and Alzheimer’s disease (Jones et al., 2011; Fu et al., 2019). Some disease-related abnormalities have not been found using stationary RSFC. Therefore, the characteristics of dRSFC have great functional significance and should be able to provide new perspectives on brain function.

Functional near-infrared spectroscopy (fNIRS) is an emerging non-invasive brain imaging technique that has great potential for evaluating the dynamic characteristics of the intrinsic brain organization. First, the time sampling rate of fNIRS can reach milliseconds (57 ms in the current study), much higher than conventional functional MRI (fMRI). In theory, better temporal resolution enables a richer temporal characterization of dRSFC with greater degrees of freedom (Zalesky et al., 2014). Second, fNIRS is not disturbed by electromagnetic fields, making it possible to be synchronously used with electroencephalography (EEG)/magnetoencephalography/fMRI technologies and allowing researchers to investigate the neural fundamentals of dRSFC. Furthermore, fNIRS can be portable, comfortable, and quiet. This facilitates fNIRS-based dRSFC applications in almost all human subjects, especially infants, various in-bed patients, and in conditions where fMRI-based dRSFC is difficult to apply (Bunce et al., 2006; Hoshi, 2007). Recently, in addition to the large number of fNIRS-based stationary RSFC studies (White et al., 2009; Lu et al., 2010; Mesquita et al., 2010; Zhang H. et al., 2010; Zhang Y. J. et al., 2010; Zhang et al., 2011; Homae et al., 2011; Duan et al., 2012; Niu and He, 2014), the feasibility of fNIRS-based dRSFC has been validated by two prior studies (Li et al., 2015; Niu et al., 2019). Specifically, based on a sliding-window correlation analysis, the variability (*Q*) of the fNIRS-based dRSFC between long-distance intrahemispheric areas (>10 cm) was found to be significantly greater than that between homotopic areas or between short-distance intrahemispheric areas (<10 cm) (Li et al., 2015), findings which are consistent with those from fMRI studies. In addition, compared with healthy subjects, it has been found that not only the global *Q* value increased in both patients with amnestic mild cognitive impairment and patients with Alzheimer’s disorder, but also there were two abnormal brain RSFC states in patients with Alzheimer’s disorder (Niu et al., 2019). However, these prior investigations of fNIRS-based dRSFC just scratched the surface. Based on fNIRS-based dRSFC, the possibility of evaluating a credible temporal evolution of a specific functional system is not very clear. With the exception of the *Q* value, other time-resolved characteristics of dRSFC, such as time-varied synchronous and antisynchronous changes between different networks, have not been investigated.

In the present study, we measured the resting-state brain fluctuations of several parts of the frontal, parietal, temporal, and occipital lobes using fNIRS. Based on a sliding-window approach and time-resolved *k*-means clustering, we extracted the evolution of dominant functional networks among these measured brain areas. We also evaluated the dynamic characteristics within a functional network, as well as the dynamic relationship between networks. Furthermore, we tried to support the credibility of fNIRS-based dRSFC analysis by exploring the relationship between the dynamic fluctuations in dRSFC networks and the switching of electrophysiological microstates based on fNIRS-EEG simultaneously recorded datasets.

## Materials and Methods

### Participants

Twenty young adults (mean age = 25.3, SD = 1.49, 11 male) were recruited from Beijing Normal University to participate in this study. No subjects had motor or other neurological diseases. Before the experiment, informed consent was obtained according to the procedure approved by the Review Board at State Key Laboratory of Cognitive Neuroscience and Learning, Beijing Normal University.

### Data Acquisition

All subjects underwent two successive resting-state sessions of fNIRS-EEG simultaneous measurements. One session was scanned with eyes closed; another was scanned with eyes open. Both resting-state sessions had a duration of 8 min. During the experiments, the subjects were seated in a chair in a silent room with dim lighting. The subjects were instructed to keep still with their eyes closed/open, relax their mind, and remain motionless as much as possible. The trigger and the terminal signs of the experiments were presented and synchronized with the fNIRS and EEG equipment using E-prime software (v.1.2, Psychological Software Tools, Pittsburgh, PA, United States). The subjects participated in these two experiments in random order. In the present study, to avoid interference from the RSFC difference between the eyes-closed and eyes-open state (Zou et al., 2009; Wong et al., 2015), we only used the eyes-closed resting-state session data to perform the dRSFC analysis.

**FIGURE 1:**
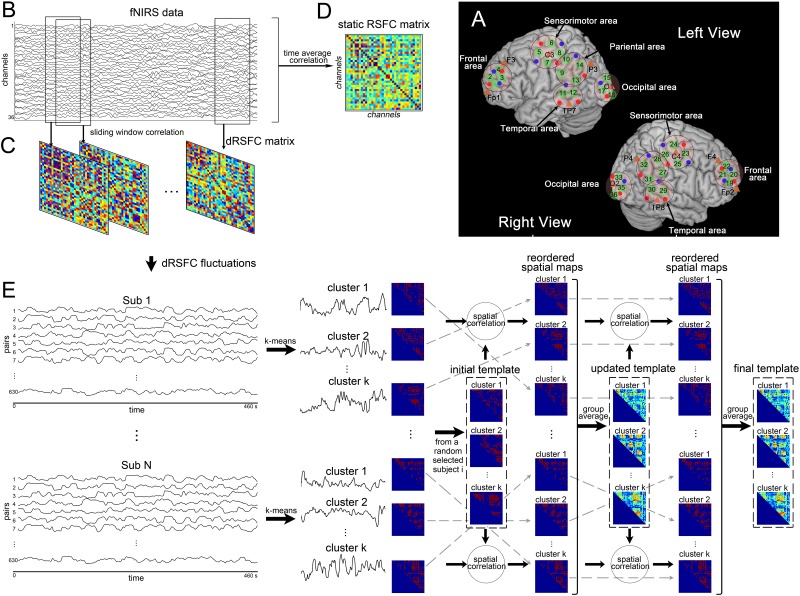
Illustration of functional near-infrared spectroscopy (fNIRS)-based dynamic resting-state functional connectivity (dRSFC) analysis. **(A)** Anatomical positions of the fNIRS standard-distance channels. Green represents the 36 fNIRS measurement channels. Red and blue dots represent the fNIRS emitters and detectors, respectively. The five ROIs were marked by black circles in the left and right views. The short-distance channels are not demonstrated here; two of these were near channels 4 and 22, and the other two were near channels 9 and 27. **(B)** An example of fNIRS data from a random selected subject. **(C)** Static RSFC analysis. The static RSFC was calculated from the time courses of the entire scanning between any two channels. **(D)** Dynamic RSFC analysis. The dRSFC was calculated between any two measurement channels using a sliding-window correlation approach. In this approach, a time window with a fixed length was selected and then shifted along the entire time course in a fixed step. Within each time window, the RSFC was calculated for each pair of measurement channels using the Pearson correlation method. This process results in quantification of time-varying dRSFC over the whole scanning. Owing to the connectivity matrix is symmetric, the effective number of connection pairs among all the 36 measurement channels is C_36_^2^ = 630. **(E)** Pipeline of clustering analysis of the dRSFC fluctuations.

fNIRS measurements were conducted with the LABNIRS fNIRS System for Research (Shimadzu Co., Kyoto, Japan). The absorption of near-infrared light at three wavelengths of 780, 805, and 830 nm was measured with a sampling time of 57 ms. The 18 emitters and 20 detector probes were plugged into a homemade holder that could combine fNIRS optodes and EEG electrodes together. The holder resulted in 40 measurement channels, including 36 standard-distance channels (interoptode distance = 30 mm) and 4 short-distance channels (interoptode distance = 15 mm). To make sure the channels could cover the regions of interest that we had previously defined, we adjusted the arrangement of the emitters and the detectors using a 3D digitizer and the registration function in NIRS-SPM software (Ye et al., 2009). With their help, we captured 3D coordinates from all the standard-distance measurement channels, transformed them to coordinates of the Montreal Neurological Institute standard template, and probabilistically estimated the structural labels as Brodmann areas according to the channels’ coordinates. Finally, we set five regions of interest (ROIs), as shown in Figure 1A. Furthermore, the four short-distance channels were located above the bilateral frontal area (near channels 4 and 22) and the temporoparietal junction (near channels 9 and 27). While considering the needs of the group analysis, the locations of the emitters and the detectors were marked according to the international 10–20 system (Jasper, 1958).

Electroencephalography data were acquired simultaneously with a 32-channel Ag–AgCl electrode cap (Neuroscan, Inc.) (international 10–20 montage and the vertical and horizontal electrooculograms). All the electrodes were referenced to the linked electrodes placed on the right mastoid. The EEG sampling rate was 1,000 Hz.

### fNIRS Data Preprocessing

For the fNIRS raw dataset, we first removed the optical density time courses before and after the experiments according to the start and the end markers set by the E-prime software. The data from one subject was removed because it lacked start/end markers (these markers were necessary for the subsequent analysis of the combined fNIRS and EEG). The concentration changes in HbO and HbR for each channel were then calculated according to the modified Beer–Lambert law (Cope and Delpy, 1988). Because several previous studies (Hoshi, 2007; Lu et al., 2010) showed that the signal/noise ratio of HbO was much higher than that of HbR, this study focused on the HbO concentration changes.

A temporal low-pass filter (<1.5 Hz) and a high-pass filter (>0.01 Hz) were applied to remove the high-frequency noise fluctuations and the ultralow frequency trends. To further remove the systemic physiological interferences arising from the superficial layer (that is, the scalp and skull), such as the vascular fluctuations arising from the cardiac pulsations, respiration, Mayer waves, and other very low-frequency fluctuations, a linear regression analysis was performed. Specifically, for each standard-distance channel, the time course was regressed the average time course for all the short-distance channels. The residual time course after regression was finally filtered again by a low-pass filter (<0.15 Hz) to eliminate the remaining high-frequency noises and retain the spontaneous brain fluctuations.

### dRSFC Calculation

For each individual, as the pipeline shown in Figures 1B,C, we estimated the dRSFC between any two measurement channels using a sliding-window correlation approach. Specifically, a 20-s time window was selected and then shifted along the entire time course in steps of one 0.057-s time point each. Within each time window, the Pearson correlation was calculated for each pair of measurement channels. Therefore, all the 36 measurement channels had an 8-min measurement duration and a 20-s sliding time window, resulting in *C*_36_^2^ = 630 pairs with 8,076 time points of dRSFC.

To compare with previous studies that focused on the spatial distribution of the variability of dRSFC, we first quantitatively estimated the variance in the dRSFC fluctuations for each pair of channels. Then, according to the five predefined ROIs, which were described above and are shown in Figure 1A, we classified the dRSFC into 15 categories, of which 5 were within ROIs and 10 were between ROIs. The mean value, as well as the standard deviation, of the variability of the dRSFC of each category was calculated. Finally, we compared the mean variability of the dRSFC between two connection groups: within the ROIs and between the ROIs, by a two-sample *t*-test.

For comparison, we also calculated the static RSFC (sRSFC). As shown in Figures 1B,D, it was proposed for each subject using a Pearson correlation analysis between any two measurement channels over the whole length of the time series. Then, to assess the sRSFC results at group level, a pair-by-pair one-sample *t-*test was performed across all the subjects and corrected by false discovery rate (FDR).

### Clustering Analysis of the dRSFC Fluctuations

To assess the temporal relationship of dRSFC between different connections, we applied a *k*-means clustering algorithm to the temporal fluctuations in the dRSFC. For each individual, we used the L2 (Euclidean) distance function during *k*-means clustering with random initialization of the centroid positions. The number of clusters (*k*) varied from 2 to 10. To eliminate the influence of random centroid positions, we repeated the clustering analyses 100 times with centroid positions updates. Then, for each *k*, a ratio (*A*) between the between-cluster distance and the within-cluster distance for each subject was computed. In our hypothesis, an acceptable value of *k* should yield greater mean values of *A* for all individuals with relatively low variance. Therefore, the final *k* was determined at the group level using the elbow criterion of the cluster validity index, defined as the ratio between the standard deviation of *A* and the mean value of *A* across all the subjects. Finally, as shown in Figure 1E, the temporal fluctuations in the dRSFC from *C*_36_^2^ = 630 pairs were classified into *k* clusters for each subject, of which the centroid positions were the time courses representing the most typical fluctuations in the dRSFC.

Because brain activity during the resting state is not time locked, the fluctuations in the RSFC (temporal structure) could not be directly compared between subjects, but their spatial distribution (spatial structure) could. Therefore, as shown in Figure 1E, we collected all the spatial distribution matrices identified for each individual subject and reordered them by two loops so we could use the same labels for the subsequent group analysis. Specifically, for the first loop, we set the *k* spatial matrices of a random selected subject as the initial template. Then, we computed a spatial correlation of the template with the individual matrices and labeled each individual matrix with the template it best corresponded to. For the second loop, the mean matrix of each cluster was calculated at the group level and set as the updated template. After repeating the above process, the individual spatial matrix was relabeled and unified at the group level.

Furthermore, to assess the transient relationship between the centroid time courses of different clusters, we quantified the instantaneous phases of each time course by a wavelet transform and calculated the time-varied phase difference between them. Specifically, we computed the convolution of the individual centroid time course of dRSFC with a complex Gabor wavelet centered at frequency *f*:

$$\text{G}\,\left(\text{t},\text{f}\right)={\text{e}}^{-\frac{{\text{t}}^{2}}{2\,{\text{σ}}_{\text{t}}^{2}}}\,{\text{e}}^{-\text{j}\,2\,\pi \,\mathrm{ft}}$$

Here, we set *f* = 0.08 Hz, σ_t_ = 10 s. The phase for this convolution was extracted for all time bins *t*:

$$\text{θ}\,\left(\text{t}\right)=\text{angle}\,\left(\text{G}\,{\left(\text{t}\right)}^{*}\,\text{x}\,\left(\text{t}\right)\right)$$

The phase difference between each of the two time courses (*x* and *y*) was defined as:

$$\Delta \,\text{θ}\,\left(\text{t}\right)=\theta_{\text{x}}\,\left(\text{t}\right)-{\text{θ}}_{\text{y}}\,\left(\text{t}\right)$$

### EEG Data Processing

First, we checked the data to remove the redundant time courses before and after the experiments according to the start and the end markers set by the E-prime software. After band-pass filtering from 1 to 100 Hz with a 50-Hz notch filter, the continuous EEG data was re-referenced with the average time course of all the effective channels. The channels with the great noise whose amplitudes were higher than five times the standard deviation of the entire time course were removed.

**FIGURE 2:**
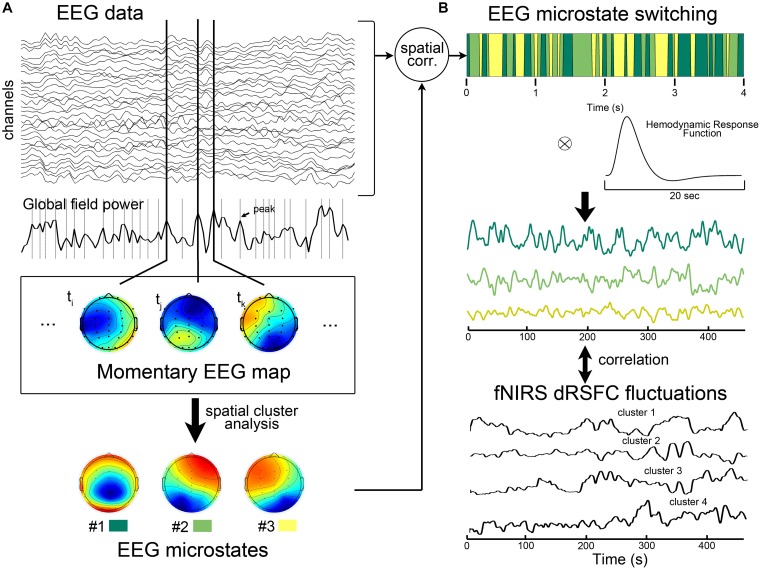
Illustration of electroencephalography (EEG)-microstate analysis and the correlation analysis between EEG microstates and fNIRS-based dRSFC fluctuations. **(A)** EEG microstate analysis. The EEG microstate was identified by a modified spatial cluster analysis from all the individual momentary EEG maps with the maxima global field powers. During this process, the optimal number of microstates was determined to be 3 based on a cross-validation criterion, which is derived by dividing the global explained variance by the degrees of freedom. **(B)** After calculating the spatial correlation between each instantaneous EEG map and the three group-level template maps, the dominant microstate was identified as the one with the greatest spatial correlation at that time point. The previous studies have shown that after the convolution of the binary time course of a EEG microstate with the hemodynamic response function, the resultant temporal smoothed signal correlated with hemodynamic fluctuations of the large-scale functional networks. Therefore, we proposed the correlation analysis between the smoothed microstate-switching signals with fNIRS-dRSFC fluctuations.

The EEG microstate was extracted according to the pipeline (introduced in Van de Ville et al., 2010). In general, we first down-sampled the EEG data to 125 Hz to reduce the computational complexity. Then, as shown in Figure 2A, we calculated the global field power index, which is the standard deviation of the potentials at all electrodes, for each time point (Brunet et al., 2011). All the time points that contained EEG global field power maxima were determined. These time points were considered to having relatively high signal/noise ratio (Brunet et al., 2011). Therefore, the momentary EEG maps at those time points were extracted as the best representative topographies. Next, using the Cartool software (v 3.55, developed by Functional Brain Mapping Lab, Geneva, Switzerland), a modified spatial cluster analysis applying the atomize-agglomerate hierarchical clustering method was used to identify the most dominant map (i.e., microstates) topographies at the individual level and at the group level. During this process, the optimal number of microstates was determined to be 3 based on a cross-validation criterion, which is derived by dividing the global explained variance by the degrees of freedom (Brunet et al., 2011). To assess the switching time course of the microstates, we calculated the spatial correlation between each instantaneous EEG map and the three group-level template maps. For each time point, the dominant microstate was selected as the one with the greatest spatial correlation. The binary time series corresponding to each microstate was considered to represent the microstate activities.

### Correlating With fNIRS-dRSFC and EEG Microstates

Some previous studies have been found that, after convolution with the hemodynamic response function, the prototypical EEG microstates during rest may have ability to explain hemodynamic activities in some specific large-scale resting state networks (Britz et al., 2010; Musso et al., 2010). Therefore, here we evaluated the relationship between the temporal fluctuations of each fNIRS-based dRSFC (the centroid time course for each cluster) and the time course of the EEG microstate switching for each subject. Specifically, as shown in Figure 2B, we first convoluted the binary time series of each EEG microstate with the canonical hemodynamic response function, which was generated by SPM toolbox. Then, the resultant smooth microstate time course was down-sampled to 17.5439 Hz, the same as the sampling rate of the fNIRS data, and correlated with the centroid time course for each fNIRS-based dRSFC cluster using the Pearson correlation index (Britz et al., 2010). Hence, we obtained a correlation matrix between all the fNIRS-based dRSFC clusters and all the EEG microstates for each subject. Using the Fisher *Z* transformation, we got a *z*-score matrix from the correlation matrix. Finally, we checked the *z*-scores at the group level using a one-sample *t*-test.

## Results

**FIGURE 3:**
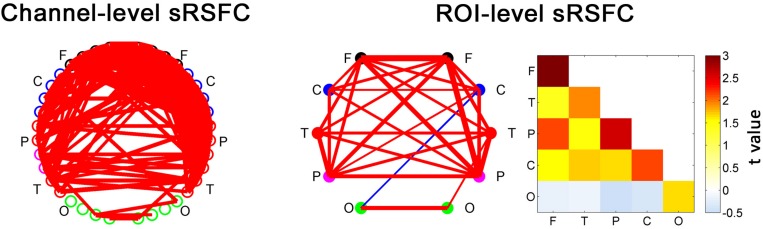
The static RSFC (sRSFC) between all the fNIRS channels with a standard interoptode distance. The sRSFCs, which are significantly greater/lower than zero at the group level (*p* < 0.01, FDR corrected), are indicated by the red/blue lines, respectively. The width of the line represents the strength of the *t*-value (one-sample *t*-test at the group level). For clarity, the ROI-level sRSFCs are shown in two forms: connectivity network map and matrix. They are the group-level statistic results after categorizing and averaging the connection edges according to an ROI partition of the nodes for each individual. F, frontal area; C, sensorimotor area; P, parietal area; T, temporal area; O, occipital area.

The sRSFCs between all the fNIRS channels with a standard interoptode distance are demonstrated in Figure 3. For clarity, the ROI-level sRSFCs are also shown in connectivity map form and matrix form. Obviously, all the connectivities between the homogeneous interhemispheric areas were significant (*p* < 0.01, FDR corrected). There were also several significant connectivities across the heterogeneous areas, but the connectivities of the occipital area were very limited.

**FIGURE 4:**
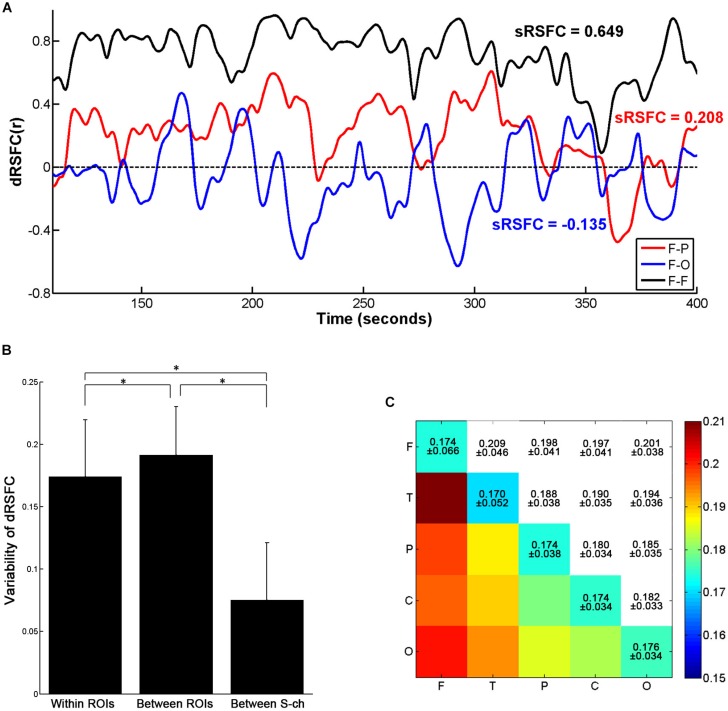
Variability of the dRSFCs. **(A)** Examples of dRSFC fluctuations for a representative subject (subject 1). The black line indicates the dRSFC between the bilateral frontal ROIs; the red line indicates the dRSFC between the frontal and the parietal ROIs; and the blue line indicates the connectivity between the frontal and the occipital ROIs. F, frontal area; P, parietal area; T, temporal area; O, occipital area. **(B)** Group comparison of variability of the dRSFC within and between ROIs and between channels with a short interoptode distance (S-ch). Error bars indicate standard deviations. Asterisks represent significant group differences with a *t*-test at *p* < 0.001 (Bonferroni corrected). **(C)** The means and standard deviations of the dRSFC variability between different ROIs at the group level.

As shown in Figure 4A, the dRSFCs between any two ROIs were highly non-stationary. Specifically, the variability of the dRSFCs within the frontal ROIs was generally great and positive at most time points. Such high values are consistent with the results from the sRSFC over the whole measurement time period (sRSFC = 0.649). By contrast, the dRSFC between the heterogeneous regions (F-P and F-O in Figure 4A) were relatively lower and exhibited both strongly positive and strongly negative correlations. The sRSFCs in these connectivities were obviously lower than those within the frontal ROIs (sRSFC = 0.208 for the F-P connectivity, and sRSFC = −0.135 for the F-O connectivity).

For the quantitative analysis, we evaluated the dRSFC variability of the connectivities within and between the ROIs. As shown in Figure 4B, the variability of the dRSFC within the ROIs was significantly lower than that between the ROIs (*p* < 0.001, Bonferroni corrected); this is consistent with the findings in previous studies (Allen et al., 2014; Li et al., 2015). In addition, by comparison with the variability in the correlation between the channels with a short interoptode distance, the variability in the dRSFC between channels with standard interoptode distances was significantly greater both within and between ROIs (*p* < 0.001, Bonferroni corrected). This implies that the dRSFC oscillations between the channels with a standard distance have some functional significance, rather than being caused by physiological noise. Specific to the connectivities between ROIs, as shown in Figure 4C, the connectivities with the greatest variability were primarily related to the frontal area, especially occurring between the frontal and the temporal areas.

**FIGURE 5:**
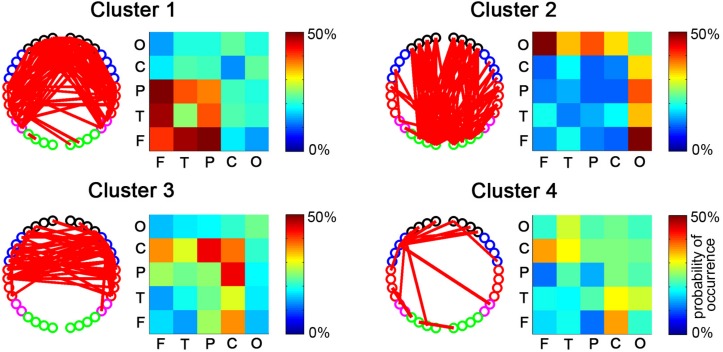
The spatial distributions of dRSFC clusters. For each cluster, the connectivity map with the dominant channel-level connectivity (left) and the ROI-level group consistency matrix (right) are demonstrated. The red lines in the left maps represent the dominant connectivity that occurred in this cluster in more than half of the participants. The values in the right matrix represent the average probability of occurrence at the group level.

After the temporal clustering analysis, four dRSFC clusters were extracted, and the probability of occurrence of each connectivity across the subjects was calculated for each cluster. For clarity, the connections for which the probability of occurrence was more than 50% are shown in the channel-level connectivity map (Figure 5). Furthermore, we averaged the probability of occurrence according to the regions to which the two nodes belong and drew the ROI-level group consistency matrix for each cluster. Obviously, cluster 1 was primarily concentrated in the connectivities between the bilateral frontal, parietal, and temporal areas. Cluster 2 was concentrated in the connections with the occipital area. Clusters 3 and 4 were primarily concentrated in the connections with the sensorimotor area, in which the first one was bilaterally symmetrical and the latter one was obviously lateralized to the left. It should be noted that, as shown in the probability of the occurrence matrix, cluster 4 had a relatively low consistency across the subjects compared with the other three clusters.

**FIGURE 6:**
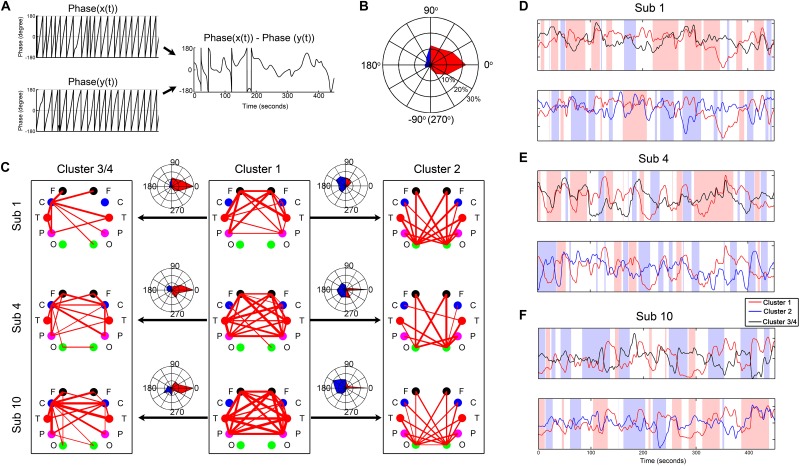
Demonstration of the instantaneous phase difference between the centroid time courses of the clusters from three representative subjects. **(A)** Illustration of the instantaneous phase difference calculation. **(B)** An example of the fingerprint map which intuitively displays the result of phase difference in **(A)**. The fingerprint map refers to the percentage of each phase difference across the whole measurement time. The three concentric circles represent 10, 20, and 30%, respectively. **(C)** Demonstration of the instantaneous phase difference between the centroid time courses of the clusters from three representative subjects. **(D–F)** The corresponding centroid time courses of the clusters from the three subjects. The pink background represents the moments with positive synchronization (−45–45°), and the blue background shows the moments with reverse synchronization (135–225°).

By a Gaber wavelet transform, we calculated the instantaneous phase difference between the centroid time courses of the four clusters. As shown in Figure 6, they constantly switched between positive correlation, negative correlation, and anticorrelation, accompanied by some system-specific preferences. For example, cluster 1 was positively synchronized with cluster 3/4 most of the time, but sometimes this was reversed. In contrast, cluster 1 was primarily negatively synchronized with cluster 2 most of the time.

**FIGURE 7:**
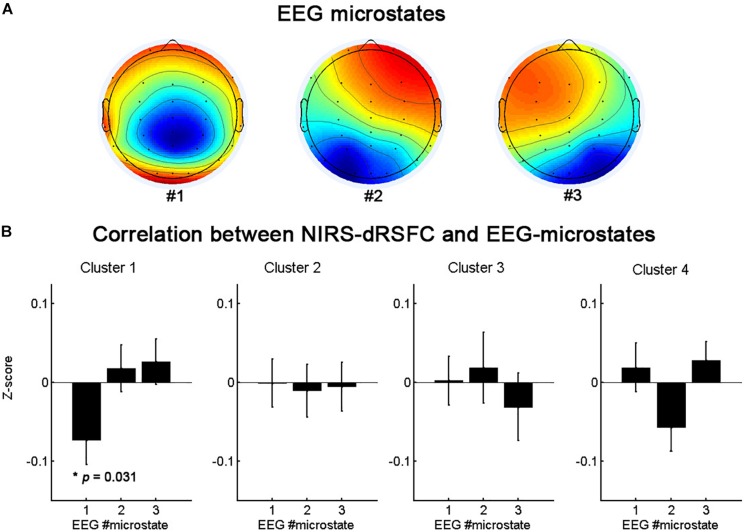
The topographies of the EEG microstates **(A)** and the statistical analysis results **(B)** for the correlation between the temporal fluctuations of fNIRS-based dRSFC clusters and the switching time course of the EEG microstates.

Figure 7 presents the topographies of the EEG microstates and the relationship between the temporal fluctuations of each fNIRS-based dRSFC (the centroid time course for each cluster) and the switching time course of the EEG microstates. The oscillation of cluster 1 was significantly correlated with the switching of the EEG microstate 1 (*p* = 0.031, uncorrected), but the other clusters did not demonstrate a significant correlation with the switching between EEG microstates.

**FIGURE 8:**
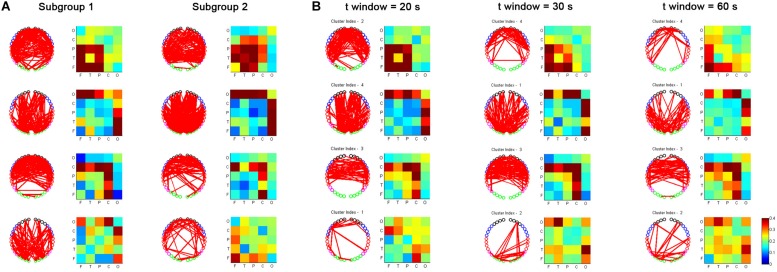
Reproducibility of fNIRS-based dRSFC from two randomly selected subgroups of subjects **(A)** and fNIRS-based dRSFC with different time window lengths (20, 30, and 60 s) **(B)**.

We also evaluated the reproducibility of the primary findings in this study. First, based on a sliding time window size of 20 s, we randomly divided the subjects into two subgroups and repeated the analysis to examine the impact of varying the subject datasets. As shown in Figure 8A, the main results, e.g., based on the temporal dRSFC clustering analysis, show that three kinds of functional networks could be extracted: the frontal-parietal-temporal network (cluster 1), the occipital network (cluster 2), and the sensorimotor network (cluster 3), keeping good consistency with the results from the entire subject groups. However, we also found that cluster 4, which had a relatively lower consistency in the entire subject group (as shown in the probability matrix of occurrence in Figure 5), also had relatively low consistency between the different subgroups. Second, using the entire subject group, we also separately used sliding time window sizes of 20, 30, and 60 s to examine the impact of varying the window lengths on the dynamic RSFC findings. The cluster results (Figure 8B) were generally consistent with the investigations reported above.

## Discussion

In this study, we first extracted the dynamic fluctuations of fNIRS-based RSFC over specific regions in the frontal, temporal, parietal, and occipital areas. Consistent with the findings in previous studies (Li et al., 2015), the variability of the dRSFC within the ROIs was significantly lower than that between the ROIs (*p* < 0.001, Bonferroni corrected), possibly resulting from relatively stable information interactions within a local region (Allen et al., 2014; Zalesky et al., 2014). The variability of the dRSFC, not only between ROIs but also within ROIs, was significantly greater than the variability in the correlation between the fNIRS channels with a short interoptode distance, an area which is located above the bilateral frontal area and the temporoparietal junction. It is known that channels with a short distance primarily consist of physiological fluctuations in the superficial layers. Therefore, this evidence implies that our fNIRS-based dRSFC oscillations have some functional significance, rather than being caused by physiological noise.

Second, by using time-resolved k-means clustering, we extracted dRSFC clusters, which were primarily focused in the three dominant functional systems. These were the frontal-parietal-temporal network, the occipital network, and the sensorimotor network. These networks are very consistent between different subject subgroups and in varying sliding time window lengths of 20, 30, and 60 s. These results confirm that the dominant functional networks could be well identified and extracted based on the time-resolved clustering of fNIRS-based dRSFC. Most importantly, we reproducibly identified the occipital network, as well as the connectivities between the occipital area and anterior regions (such as the frontal area). Such connections are critical for visual object processing (Sehatpour et al., 2008) and consciousness (Giacino et al., 2014; Koch et al., 2016) but may not be revealed by static fNIRS-based RSFC (Mesquita et al., 2010). As reported in previous works, the most dynamic connections always correspond to the weakest stationary RSFC (Li et al., 2015) because the dynamic RSFCs are near zero in the time averages. Thus, it is apparent that, since analyses using the stationary assumption reduce the information about RSFC fluctuations to time averages, these convenient RSFC analyses may have led to an oversimplified characterization of the brain’s functional networks. The successful extraction of the occipital network in the present study suggests that assessing functional connectivity from their dynamic characteristics could rectify the deficiency of the static fNIRS-based RSFC by identifying some “hidden” functional connectivity/system.

We also evaluated the synchrony between the different functional networks and found how the synchrony varied through time. The networks constantly switch between positive correlation, negative correlation, and anticorrelation, accompanied by some system-specific preferences. The frontal-parietal-temporal network (cluster 1), for example, was primarily positively synchronized with the sensorimotor network (cluster 3/4) and negatively synchronized with the occipital network (cluster 2) during most of the time intervals, but sometimes this was reversed. This finding is consistent with a previous fMRI-based dynamic RSFC analysis that found that the temporal fluctuations of the dRSFC were an essential property that can unveil flexibility in the dynamic functional coordination between different neural systems (Allen et al., 2014). In particular, based on time-resolved methodologies for analyzing the variability of the dRSFC, most dynamic connectivities were found to be intermodular (i.e., to link elements from the separable subsystems) and localized to known hubs, such as the default mode regions, superior occipital networks, and fronto-parietal systems (Allen et al., 2014; Zalesky et al., 2014). Their alternating pattern of correlations and anticorrelations implies that these intermodular regions may be connected with specific systems for a fraction of the time. That is, the multiple functional associations among these systems are realized through a dynamic process of time-division multiplexing (Zalesky et al., 2014).

Similar to the dynamic functional connectivity of brain hemodynamic activation, the momentary global state of the neurophysiological activities does not change randomly and continuously over time (Lehmann et al., 1987; Van de Ville et al., 2010). These momentary global states, termed “EEG microstates,” remain stable for ∼80–120 ms and can be assessed by analyzing the temporal evolution of the EEG scalp topography. With a limited number of prototypical configurations, EEG microstates are persistently identified across the entire life span (Keilholz et al., 2013). The changing of the EEG microstates in the resting state indicates that rapid switching between the activities of the neural assemblies of the brain occurs. Research has showed that, after convolution with the hemodynamic response function, the rapidly fluctuating EEG microstates correlate significantly with the slow oscillations of fMRI resting-state networks (Britz et al., 2010; Musso et al., 2010; Yuan et al., 2012). In the present study, to further support the credibility of the dRSFC data and to explore the neural basis of the RSFC dynamic fluctuation, we evaluated the relationship between the temporal fluctuations of each fNIRS-based dRSFC (the centroid time course of each cluster) and the switching time course of EEG microstates based on simultaneous recording fNIRS-EEG datasets. We found that the oscillation of the frontal-parietal-temporal network (cluster 1) was significantly correlated with the switching of the EEG microstate 1. The spatial distributions of the these three EEG microstates are generally consistent with the map in the previous EEG microstate studies (Britz et al., 2010; Khanna et al., 2015). EEG microstate 1 roughly corresponded to map 4 in Britz et al., 2010, which was found to be significantly correlated with the hemodynamic activity in a right-lateralized dorsal frontoparietal network, mainly contributing to switching and reorienting attention. It is consistent with the findings in the present study, implying the credibility of fNIRS-based dRSFC networks.

In previous fMRI-based dRSFC studies, the dorsal attention areas and default-mode regions were consistently assigned to the partition with a more variable connectivity (Allen et al., 2014; Zalesky et al., 2014). In our study, however, the connections with the greatest variability were primarily related to the frontal area, especially between the frontal and the temporal areas. We guess that this discrepancy may have result from the relatively better signal/noise ratio in the frontal area during the fNIRS data acquisition. This finding reminds us that the variability analysis in fNIRS-based dRSFC could be influenced by the signal/noise ratio of the data. Future research needs to be careful to avoid this disruptive factor.

In this study, although clusters 1–3 were highly consistent; cluster 4 had a relatively lower consistency between the different subject subgroups, within any subject group, or across different time window lengths. We are not sure about the exact reason but speculate that this may have been due to uncontrollable behavior and mental diversity between individuals and at different time points. This may also have resulted from the diversity of the spatial localization of the fNIRS measurement channels between subjects. This needs to be further investigated in future studies with a larger sample size and repeated measurements.

## Conclusion

In conclusion, this study demonstrated the functional significance of fNIRS-dRSFC and the feasibility of fNIRS for extracting the dominant functional networks based on RSFC dynamics. Because of the advantages of fNIRS in clinical applications, fNIRS-based dRSFC research could help researchers gain insight into the relationship between time-varying brain activity patterns and critical aspects of cognition and behavior by making up for the deficiencies of stable RSFC research.

## Data Availability Statement

The datasets generated for this study are available on request to the corresponding author.

## Ethics Statement

The studies involving human participants were reviewed and approved by the Review Board at State Key Laboratory of Cognitive Neuroscience and Learning, Beijing Normal University. The participants provided their written informed consent to participate in this study.

## Author Contributions

YZ and CZ contributed to the conception and design of the study. YZ organized the database, performed the analysis, and wrote the first draft of the manuscript. CZ contributed to the manuscript revision.

## Conflict of Interest

The authors declare that the research was conducted in the absence of any commercial or financial relationships that could be construed as a potential conflict of interest.
